# Improving molecular diagnosis of aniridia and WAGR syndrome using customized targeted array-based CGH

**DOI:** 10.1371/journal.pone.0172363

**Published:** 2017-02-23

**Authors:** Fiona Blanco-Kelly, María Palomares, Elena Vallespín, Cristina Villaverde, Rubén Martín-Arenas, Camilo Vélez-Monsalve, Isabel Lorda-Sánchez, Julián Nevado, María José Trujillo-Tiebas, Pablo Lapunzina, Carmen Ayuso, Marta Corton

**Affiliations:** 1 Department of Genetics & Genomics, Instituto de Investigación Sanitaria-Fundación Jiménez Díaz University Hospital- Universidad Autónoma de Madrid (IIS-FJD, UAM), Madrid, Spain; 2 Centre for Biomedical Network Research on Rare Diseases (CIBERER), ISCIII, Madrid, Spain; 3 Institute of Medical & Molecular Genetics (INGEMM), Hospital Universitario La Paz, Universidad Autónoma de Madrid, IdiPAZ, Madrid, Spain; Queen Mary Hospital, HONG KONG

## Abstract

Chromosomal deletions at 11p13 are a frequent cause of congenital Aniridia, a rare pan-ocular genetic disease, and of WAGR syndrome, accounting up to 30% of cases. First-tier genetic testing for newborn with aniridia, to detect 11p13 rearrangements, includes Multiplex Ligation-dependent Probe Amplification (MLPA) and karyotyping. However, neither of these approaches allow obtaining a complete picture of the high complexity of chromosomal deletions and breakpoints in aniridia. Here, we report the development and validation of a customized targeted array-based comparative genomic hybridization, so called WAGR-array, for comprehensive high-resolution analysis of CNV in the WAGR locus. Our approach increased the detection rate in a Spanish cohort of 38 patients with aniridia, WAGR syndrome and other related ocular malformations, allowing to characterize four undiagnosed aniridia cases, and to confirm MLPA findings in four additional patients. For all patients, breakpoints were accurately established and a contiguous deletion syndrome, involving a large number of genes, was identified in three patients. Moreover, we identified novel microdeletions affecting 3' *PAX6* regulatory regions in three families with isolated aniridia. This tool represents a good strategy for the genetic diagnosis of aniridia and associated syndromes, allowing for a more accurate CNVs detection, as well as a better delineation of breakpoints. Our results underline the clinical importance of performing exhaustive and accurate analysis of chromosomal rearrangements for patients with aniridia, especially newborns and those without defects in *PAX6* after diagnostic screening.

## Introduction

Congenital aniridia [MIM#106210] is a rare genetic pan-ocular disease characterized by the complete or partial absence of the iris. It can appear uni- or bilaterally, isolated or in association to other ocular and/or systemic anomalies. Affected individuals can present additional congenital or progressive ocular anomalies [1, 2]. Among syndromic cases with aniridia, the most frequent form is the WAGR syndrome [MIM#194072, Wilms Tumor, Aniridia, Genitourinary anomalies and mental Retardation] [3]. In most of case, aniridia and related-syndromic conditions are caused by heterozygous loss-of-function defects in *PAX6*, located on chromosome 11p13. Missense mutations in *PAX6* have been also associated with other isolated ocular anomalies [1, 2, 4]. Up to 30% of patients with aniridia carry chromosomal rearrangements at 11p13, with a high degree of breakpoint complexity [5, 6], including whole-gene deletions, microdeletions affecting only 3´ regulatory enhancers [5, 7–12] or contiguous gene deletions of *PAX6* and other neighboring genes, mainly *WT1* which is associated to WAGR syndrome [1, 13].

Due to the high complexity of the genetic mechanisms involved in congenital aniridia and related syndromes, clinical testing is currently performed by combining different molecular and cytogenetic approaches, such as Sanger sequencing for screening of intragenic *PAX6* mutations, Multiplex Ligation-dependent Probe Amplification (MLPA) and/or Fluorescence In Situ Hybridization(FISH) for analysis of small interstitial 11p13 microdeletions, and conventional or high resolution karyotyping for other microscopic 11p13 rearrangements [6, 14–16]. MLPA allows for a rapid and quite reliable analysis of targeted *PAX6* and *WT1* deletions [17]. However, there are some constraints underlying the specific design of commercial MLPA kits, which clearly limit its application to the identification of only some types of rearrangements. Beside, MLPA does not allow for a determination of CNVs boundaries and mapping of breakpoints. Solving those limitations is crucial for the correct diagnostic of deletions involving the *PAX6* region in newborns with sporadic aniridia, in which a 11p13 deletion analysis should be prioritized to discard WAGR syndrome [4, 13].

Nowadays, high-resolution array-based comparative genomic hybridization (array-CGH) has proven to be a more sensitive and cost-effective approach for copy number variations (CNVs) analysis than conventional cytogenetic and MLPA techniques. Thus, they are extensively used as a diagnostic tool for detecting large CNVs in patients presenting development disabilities and / or birth defects [18]. Besides, several authors have also established effectiveness of microarray analysis for the study of congenital ocular malformations [19–21]. However, few studies have been aimed at the genomic analysis of 11p13 microdeletions in large case-series of aniridia and/or related syndromes by high-resolution array-CGH [22–24].

Gene or exon-targeted arrays can now be easily developed with probes densely distributed across individual genes, allowing to explore small intragenic CNVs at the resolution of single exons [25, 26]. Beside, these specific high-resolution arrays not only optimize the accurate identification of smaller CNVs but also have a better performance for fine mapping of breakpoints [27, 28]. Single *locus* arrays have been developed in recent years to detect exonic and intronic CNVs for different genetic diseases, such as Duchenne muscular dystrophy, cystic fibrosis, retinal dystrophies and oculocutaneous albinism [26, 29–31]. Herein, we report the use of a customized array-CGH with high-density probe coverage of the 11p13 region, to study chromosomal rearrangements in the WAGR locus. Our study demonstrates that this experimental approach complements routine clinical analysis, allowing for a more accurate and refined molecular diagnosis of previously known 11p13 microdeletions and increasing the detection rate of chromosomal rearrangements in a cohort of uncharacterized cases with aniridia.

## Materials and methods

### Patients

Our study cohort consisted of 38 unrelated Spanish cases with congenital ocular malformations that had been referred to the Fundación Jiménez Díaz University Hospital (FJD, Madrid, Spain) for the genetic study of *PAX6*. Nineteen families had been diagnosed with isolated or syndromic aniridia, including: i) 13 patients without intragenic *PAX6* mutations after Sanger sequencing; ii) four patients carrying a previously known 11p13 microdeletion detected by MLPA, that were used as positive controls for validation purposes, and iii) two cases carrying a coding *PAX6* mutation, that were used as negative control samples. Additionally, 19 patients with a non-aniridia eye developmental disorder were also studied, consisting of 10 individuals with ocular coloboma, seven patients with anterior segment dysgenesis and two patients with optic nerve anomalies. Written informed consent was obtained from all subjects or their legal guardians prior to their participation in this study. This study was reviewed and approved by the Ethics Committee of the Fundacion Jimenez Diaz University Hospital and it was performed according to the tenets of the Declaration of Helsinki and further reviews.

### Genetic analysis

Genomic DNA was extracted from peripheral blood samples using automated DNA extractors (BioRobot EZ1, QIAGEN, Hilden, Germany and / or MagnaPure, Roche Diagnosis, Basel, Switzerland). Previously to this study, genetic analysis of *PAX6* was performed following our previously established diagnostic algorithm [4, 13]. Mutations in coding regions of *PAX6* were analyzed by Sanger sequencing. CNVs affecting *PAX6* and *WT1* were studied using commercial MLPA kits (Salsa P219; MRC Holland, Amsterdam, the Netherlands), following the manufacturer’s protocols. Amplification products were separated on an ABI3130xl sequencer and analyzed using the GeneMapper software (Applied Biosystems, Foster City, CA, USA). MLPA analysis was performed using the Coffalyser software (MRC Holland).

### Microarray experiments

#### Array design

A customized oligonucleotide chip targeting 5 Mb in the *PAX6* and *WT1* region (chr11:29,750,000–34,749,999) was specifically designed. The features were selected from Agilent eArray webtool (Agilent Technologies, Santa Clara, CA, USA; https://earray.chem.agilent.com/earray) probe library in a custom high-resolution format of 8x15K that contains 15,744 distinct biological 60-mer oligonucleotides probes. Thus, a total of 616 oligonucleotide probes cover *PAX6* with an average probe spacing of 54 bp, 674 oligonucleotide probes cover *WT1* with an average probe spacing of 71 bp and in the rest of 5 Mb region, the average probe spacing is 490 bp. Additional probes were homogeneously distributed as backbone probes outside the target region in chr11 and the remaining chromosomes. The probe sequences and gene annotation are based on NCBI Build 37/UCSC version hg19. Arrays were manufactured with Agilent’s Sure-Print Inkjet technology (Agilent Technologies).

#### Microarray processing

Array experiments were performed as recommended by the manufacturer (Agilent Technologies). DNAs (500 ng) from the specimen and a sex-matched reference (Promega, Madison, WI, USA) were double-digested with RsaI and AluI for 2h at 37°C. After heat inactivation of the enzymes at 65°C for 20 min, each digested sample was labelled by random priming (Genomic DNA Enzymatic Labelling Kit, Agilent) for 2 h using Cy5-dUTP for patient DNAs and Cy3-dUTP for reference DNAs. Labelled products were column-purified using Microcon Ym-30 filters (Merck Millipore Corporation, Darmstadt, Germany). Hybridization was performed at 65°C with rotation for 24 h. After two washing steps, the array was analyzed with the Agilent scanner using the Feature Extraction software (Agilent Technologies).

#### Data analysis

The analysis and visualization of array data were performed using Genomic Workbench Standard Edition software (Agilent Technologies). Comprehensive description of the statistical algorithms is available in the user’s manual provided by Agilent Technologies. The Aberration Detection Method 2 (ADM-2) quality weighted interval score algorithm identifies aberrant intervals in samples that have consistently high or low log ratios based on their statistical score. The score represents the deviation of the weighted average of the normalized log ratios from its expected value of zero calculated with Derivative Log2 Ratio Standard Deviation algorithm. A Fuzzy Zero algorithm is applied to incorporate quality information about each probe measurement. To make a positive call, our threshold settings for the CGH analytics software were 6.0 for sensitivity, 0.35 for minimum absolute average log ratio per region. Three consecutive probes with the same polarity were required for the minimum number of probes per region. All arrays were scanned at 3 μm resolution (Agilent Technologies).

## Results

### Validation of a custom CGH array for aniridia and WAGR syndrome

First, we have evaluated the WAGR-array performance by means of positive and negative control samples. Two negative control samples, carrying point mutations previously identified by Sanger sequencing, gave normal results on the WAGR array. Four samples from isolated aniridia or WAGR patients with known 11p13 deletions previously identified by MLPA (ANIRIDIA-020, ANIRIDIA-039, ANIRIDIA-064 and ANIRIDIA-067; Table 1) were used as positive controls; they ranged from a small deletion, affecting apparently only two exons of *PAX6*, to large rearrangements encompassing multiple genes. All four CNVs were correctly identified and a larger deletion was clearly identified, resulting in a more accurate definition of breakpoints. Thus, neither false-negative nor false-positive results were observed.

**Table 1:** Patients with isolated or syndromic aniridia carrying chromosomal deletions in 11p13.

*Family*	*Inheritance*	*Phenotype*	*Findings in previous testing*	*Cytogenetic band*	*Genomic coordinates (hg19)*	*Involved genes*	*Size*
**ANIRIDIA-008**	AD	Isolated aniridia	Negative	11p13	chr11:31,147,306–31,714,853	*DCDC1*, *DNAJC24*, *IMMPL1*, *ELP4*	567 Kb
**ANIRIDIA-020**	de novo	WAGR	Deletion *PAX*6 and *WT1* by MLPA	11p14.1-11p13	chr11:29,750,813–32,752,091	*KCNA4*, *FSHB*, *C11orf46*, *ARL14EP*, *MPPED2*, *DCDC5*, *DCDC1*, *DNAJC24*, *IMMP1L*, *ELP4*, ***PAX6***, *RNC1*, ***WT1***, *EIF3M*, *CCDC73*	3 Mb
**ANIRIDIA-021**	de novo	Isolated aniridia	Negative	11p13	chr11:31,186,493–31,698,208	*DCDC1*, *DNAJC24*, *IMMPL1*, *ELP4*	512 Kb
**ANIRIDIA-039**	de novo	Isolated aniridia	Deletion of exon 6 and 7 of *PAX6* by MLPA	11p13	chr11:31,820,789–31,824,052	***PAX6*** (exons 5a, 6 and 7)	3.3 Kb
**ANIRIDIA-052**	AD	Isolated aniridia	Negative	11p13	chr11:31,760,458–31,823,847	***PAX6*** (exons 5a to 13) and *ELP4*	63 Kb
**ANIRIDIA-064**	de novo	Syndromic aniridia	Deletion of 4 Mb, including *PAX6*, *ELP4*, *DCDC1*, *FSHB and BDNF* by MLPA.	11p15.1-11p13	chr11:18,536,224–31,923,308	*TSG101*, *UEVLD*, *SPTY2D1*, *TMEM86A*, *IGSF22*, *PTPN5*, *MRGPRX1*, *MRGPRX2*, *ZDHHC13*, *CSRP3*, *E2F8*, *NAV2*, *LOC100126784*, *DBX1*, *HTATIP2*, *PRMT3*, *SLC6A5*, *NELL1*, *ANO5*, *SLC17A6*, *FANCF*, *GAS2*, *SVIP*, *LUZP2*, *ANO3*,*MUC15*, *SLC5A12*, *FIBIN*, *BBOX1*, *CCDC34LGR4*, *LIN7C*, *BDNFOS*, ***BDNF***, *KIF18A*, *MIR610*, *METT5D1*, *KCNA4*, *FSHB*, *C11orf46*, *MPPED2*, *DCDC1*, *DNAJC24*, *IMMP1L*, *ELP4*, ***PAX6*,** *RCN1*	13.9 Mb
**ANIRIDIA -067**	AD	Isolated aniridia	Deletion of *DCDC1* and *ELP4* by MLPA	11p13	chr11:31,083,877–31,704,548	*DCDC1*, *DNAJC24*, *IMMPL1*, *ELP4*	620 Kb
**ANIRIDIA -070**	de novo	Syndromic Aniridia (WAGRO)	Negative	11p14.3-11p13	chr11:21,586,131–33,168,232	*NELL1*, *ANO5*, *SLC17A6*, *FANCF*, *GAS2*, *SVIP*, *LUZP2*, *ANO3*, *MUC15*, *SLC5A12*, *FIBIN*, *BBOX1*, *CCDC34*, *LGR4*, *LIN7C*, *BDNFOS*, *BDNF*, *KIF18A*, *MIR610*, *METT5D1*, *KCNA4*, *FSHB*, *C11orf46*, *MPPED2*, *DCDC1*, *DNAJC24*, *IMMP1L*, *ELP4*, ***PAX6***, *RNC1*, ***WT1***, *WT1-AS*, *EIF3M*, *CCDC73*, *TCP11L*, *QSCR*, *DEPDC7*, *CSTF3*	11.6 Mb

AD: Autosomal Dominant. MLPA: Multiplex Ligation-dependent Probe Amplification. WAGR: WAGR syndrome (Wilms Tumor, Aniridia, Genitourinary anomalies and mental Retardation). WAGRO: WAGRO syndrome (WAGR + Obesity). The *PAX6* and *WT1*genes, implicated in aniridia and WAGR syndrome, and the *BDNF* gene, implicated in WAGRO syndrome, are indicated in bold.

**Fig 1:**
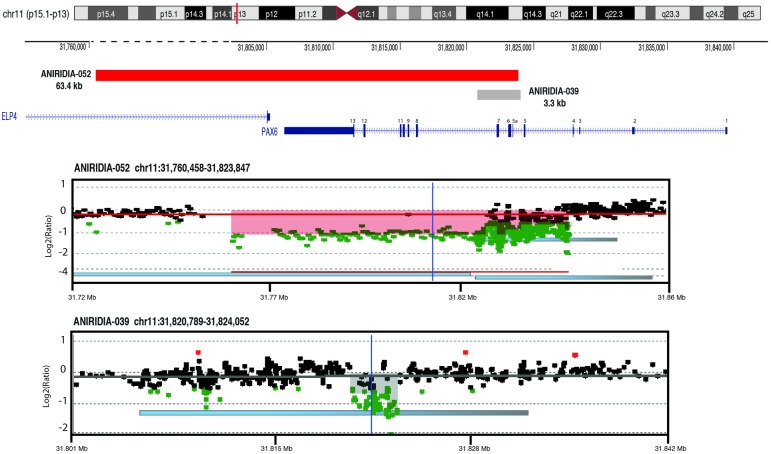
Identification of intragenic *PAX6* deletion in patients with isolated aniridia. Targeted array-based comparative genomic hybridization (aCGH) analysis identified two deletions involving partial *PAX6* deletions in two patients. Colored bars represent the genomic positions of the deletions. Schematic representation of the complete intron-exon structure of *PAX6* is shown. Exons are indicated by colored rectangles that are wider for the coding regions. CGH array data for both individuals is shown. The patient *versus* reference log2-ratio for the relative hybridization intensities of probes is plotted. Dots with log2-ratio around -1 indicate a heterozygous deletion (green dots), log2-ratio 0 indicates a normal pattern, and +0.6 indicates a heterozygous amplification (red dots). Shaded areas indicate deletions. Genomic coordinates are shown in the x-axis and are based on the Human Genome Assembly hg19. The red bar indicates a ~63 kb deletion encompassing from exon 5a to exon 13 of *PAX6* found in patient ANIRIDIA-052 (chr11:31,760,458–31,823,847). The grey bar represents a ~3.3 kb deletion encompassing from exon 5a to exon 7 of *PAX6* gene found in patient ANIRIDIA-039 (chr11:31,820,789–31,824,052).

**Fig 2:**
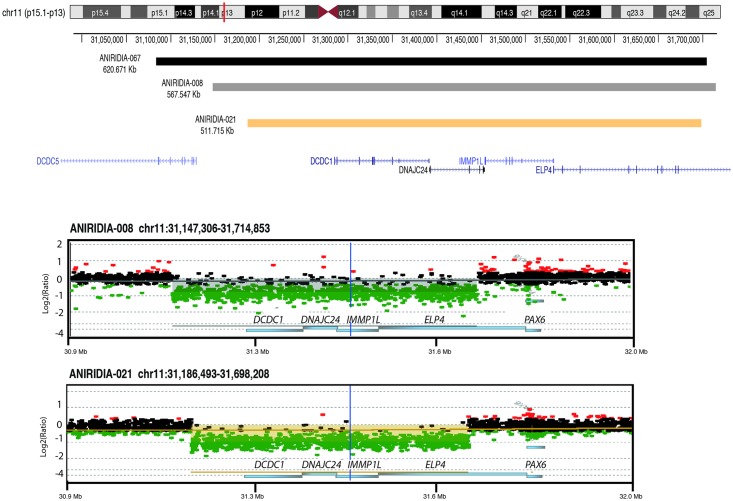
Identification of 3’ regulatory deletions of *PAX6* in patients with isolated aniridia. Targeted array-based comparative genomic hybridization (aCGH) analysis identified deletions involving telomeric deletions to *PAX6* in two patients ANIRIDIA-008 and ANIRIDIA-021. Patient ANIRIDIA-067 was used as positive control for validation purposes. The colored bars represent the genomic positions of the deletions. The red asterisks indicate a cluster of *PAX6* regulatory regions located in intronic positions of *ELP4*, as reviewed by Bathia, *et al*, 2013. Exons are indicated by colored rectangles that are wider for the coding regions. CGH array data for the two patients with previously unknown 3' telomeric *PAX6* deletions are shown. The patient *versus* reference log2-ratio for the relative hybridization intensities of probes is plotted. Dots with log2-ratio around -1 indicate a heterozygous deletion (green dots), log2-ratio 0 indicates a normal pattern and +0.6 indicates a heterozygous amplification (red dots). Shaded areas indicate deletions. Genomic coordinates are shown in the x-axis and are based on the Human Genome Assembly hg19. The grey bar indicates a ~567 kb deletion in patient ANIRIDIA-008 (chr11:31,147,306–31,714,853). The orange bar indicates a ~511 kb deletion in patient ANIRIDIA-021 (chr11:31,186,493–31,698,208).

This way, the smallest deletion found in controls was a *de novo* heterozygous 3.3 Kb deletion of three exons (exon 5a, 6 and 7) of *PAX6* in a sporadic case of isolated aniridia (ANIRIDIA-039), that had been thought to affect only exons 6 and 7 by MLPA analysis. WAGR array allowed detecting a bigger CNV affecting also the exon 5a, an alternative exon located in intron 5 of *PAX6* (Table 1, Fig 1). We also analyzed an affected individual with isolated aniridia from an autosomal dominant family (ANIRIDIA-067) that apparently carried a 3' intergenic deletion. This was defined by MLPA analysis as a dosage reduction for three peaks of *ELP4* and *DCDC1*, two adjacent genes located downstream to *PAX6*. WAGR array refined the deletion as a 620 Kb loss located 3' to *PAX6*, encompassing 5 genes (*ELP4*, *IMMPL1*, *DNAJC24*, *DCDC1 and DCDC5*) (Table 1, Fig 2). We confirmed that this deletion did not affect the structural *PAX6* region but a *PAX6* cluster of transcriptional regulatory elements, located in intronic regions of *ELP4* [8, 32]. Further analysis confirmed that this aberration correctly segregated with an autosomal dominant pattern.

**Fig 3:**
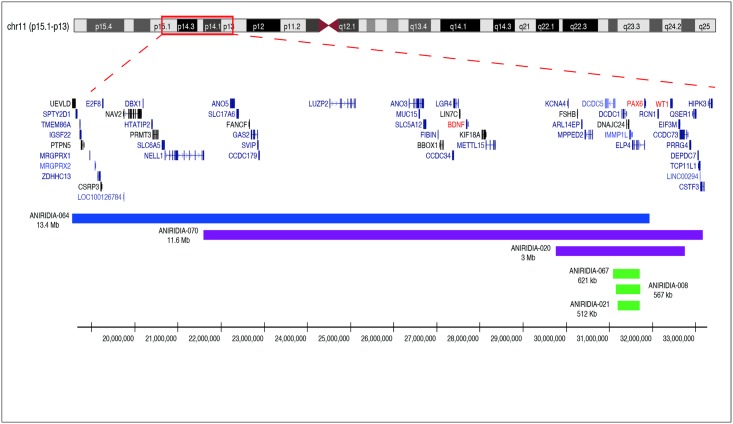
Identification of chromosomal rearrangements in WAGR locus in syndromic and non-syndromic patients with aniridia. Targeted array-based comparative genomic hybridization (aCGH) analysis identified deletions of different sizes ranging from3.3 Kb to 13.4 Mb. The red bars show intragenic *PAX6* deletions in two patients with isolated aniridia, ANIRIDIA-039 (chr11:31,820,789–31,824,052) and ANIRIDIA-052 (chr11:31,760,458–31,823,847). The green bars show 3’ upstream deletions affecting 3' regulatory regions of *PAX6*, identified in three families with isolated aniridia: ANIRIDIA-008 (chr11:31,147,306–31,714,853), ANIRIDIA-021 (chr11:31,186,493–31,698,208) and ANIRIDIA-067 (chr11:31,083,877–31,704,548). Purple bars show large deletions affecting several contiguous genes, in two patients with WAGR (ANIRIDIA-020, chr11:29,750,813–32,752,091), and WAGRO (ANIRIDIA-070, chr11:21,586,131–33,168,232) syndromes. Genes delineating both syndromes are highlighted in red. The blue bar represents a novel gene contiguous deletion syndrome involving *PAX6* and 45 upstream genes in a syndromic patient with aniridia (ANIRIDIA-064, chr11:18,536,224–31,923,308). Genomic coordinates are shown in the x-axis and are based on the Human Genome Assembly hg19.

We also tested as positive control two large contiguous gene deletions on 11p13. Both were correctly identified by the WAGR array. We included a patient clinically and molecularly diagnosed of WAGR syndrome (ANIRIDIA-020) to evaluate larger rearrangements affecting *WT1*. In this patient, MLPA analysis had shown a complete deletion of the *PAX6*, *WT1*, *ELP4* and *DCDC1* genes. WAGR array identified a larger deletion of 3 Mb, affecting 11 additional neighboring genes (Table 1, Fig 3). Finally, we included a sporadic patient with syndromic aniridia (ANIRIDIA-064) in which MLPA analysis had allowed identifying a deletion of at least 4 Mb affecting *PAX6* and several neighboring genes, including *ELP4*, *DCDC1*, *FSHB* and *BDNF*. Interestingly, *WT1* had seemed not to be deleted; thus WAGR syndrome had been excluded in this patient. WAGR-array allowed us to confirm this suspicion by delimiting the proximal breakpoint located around 534 Kb upstream of *WT1*. In addition, a larger deletion of 13.4 Mb was refined at 11p15.1-11p13 encompassing 47 genes and extending outside the targeted region (Table 1, Fig 3).

### Identification of novel rearrangements affecting 11p13

We used our custom WAGR array as a second-line diagnostic method to test 32 additional patients: 12 cases with isolated aniridia, one syndromic patient with aniridia and 19 patients with several other ocular malformations that could potentially be caused by *PAX6* anomalies. Except one patient with isolated aniridia carrying a previously known translocation tr(6,11), all of them were uncharacterized patients for which conventional *PAX6* testing by Sanger sequencing and MLPA were negative or inconclusive. In four patients, we identified deletions affecting 11p13, sizing from 63Kb to 11Mb and involving *PAX6*, 3’ regions and/or neighboring genes (Table 1). We only identified CNVs in patients with a diagnosis of aniridia; thus, none of the 19 patients suffering from other ocular phenotypes showed altered patterns on the WAGR-array.

In two uncharacterized patients with isolated aniridia (ANIRIDIA-008 and ANIRIDIA-021), we identified deletions affecting 3’ regions to *PAX6*, sizing 512 and 567 Kb, respectively. Both deletions encompassed *DCDC1*, *DNAJC24*, *IMMPL1* and *ELP4*, without involvement of the *PAX6* genomic region (Fig 2). A subsequent analysis with an updated MLPA version, containing three probes for this region, allowed us to confirm these findings. Additionally, in a familial case of isolated aniridia (ANIRIDIA-052), the WAGR-array found a microdeletion of 63 Kb affecting exons 5a to 13 of *PAX6*, and also partially of the downstream *ELP4* gene (Fig 1). Interestingly, previous MLPA analysis had not detected peak anomalies of the specific probes for these exons, thus this case may represent a false-negative of this technique. Further segregation in the affected mother allowed us to confirm this CNV. The fourth newly identified patient (ANIRIDIA-070) was a sporadic case of aniridia with developmental delay, in which WAGR-array evidenced the presence of a large deletion of 11.6 Mb (Table 1, Fig 3), affecting 38 genes and including *PAX6*, *WT1*and *BDNF*, this last gene is responsible for the obesity in the WAGRO syndrome [MIM#612469; WAGR with Obesity]. Therefore, this patient was diagnosed as being at risk of developing WAGRO syndrome.

In sum, our custom aCGH array showed a diagnostic rate of 31% (4/13) for uncharacterized cases with aniridia, and an overall detection rate of 42% (8/19) in the complete aniridia cohort analyzed, including controls. No clinically pathogenic CNVs were detected in additional patients with other *PAX6*-related ocular diseases.

## Discussion

Nowadays, it is important to exhaustively explore the non-coding regions of *PAX6* by mean of more robust and sensitive methods for CNVs detection that the routinely used in the study of aniridia. In this way, the percentage of patients with aniridia that appear not to carry *PAX6* defects after routine genetic analysis could be probably reduced.[4].

For CNVs screening in clinical routine, commercial chromosomal microarrays (CMA) or targeted array-CGH, specifically designed to screen clinically relevant regions, are the recommended diagnostic assays when no specific locus is suspected [28]. They should be designed to detect genomic anomalies of >100–200 Kb with a very high sensitivity and specificity [18, 33]. Depending on the platform, coverage design and spacing of probes of these commercial designs, the resolution level could not be optimal to screen smaller CNVs in single-gene disorders, such as Aniridia. Although commercial CMAs could accurately detect large deletions of several Mb in the WAGR locus or even 3’ *PAX6* deletions of around 500 kb, as demonstrated by different studies [11, 12, 22, 24, 34], most of them do not contain enough probe coverage for the WAGR locus to detect CNVs at the scale of exons. Thus, they can fail in the detection of smaller microdeletions affecting single or multiple exons, or any of the enhancers elements of *PAX6*. In addition, they only let for a rough determination of breakpoint boundaries.

Gene or exon-targeted arrays currently represent a very cost-effective, reliable and sensitive approach to detect small intragenic CNVs at very high-resolution level in the interest regions, having a great flexibility through customized designs [35]. Here, we propose the use of a custom single-locus array-CGH for anirida, so-called WAGR-array, that allows an accurate high-resolution CNVs detection in the WAGR locus at 11p13-14. Unlike other exon-level designs which do not usually include probes for intronic and regulatory regions [23], WAGR-array makes possible a reliable and unambiguously identification of CNVs at several levels of resolution from large genomic imbalances to very small deletions of individual exons. In fact, we were able to find a small CNV of only 3 Kb involving three exons of *PAX6*. As this is the smaller rearrangement available in our series, it could be considered as the sensitivity threshold for our study. Because our design has a theoretically minimum average resolution of 160 bp for intragenic PAX6 regions, it is expected that its detection threshold may be potentially lower. Besides, as the genomic region surrounding *PAX6* are also highly covered, with an average resolution of 1.5 kb, WAGR-array also allows accurately identifying and delimiting 3' *PAX6* deletions, as demonstrated in three families with sporadic aniridia. In addition, the inclusion of additional backbone probes covering the rest of chr11 also allows exploring large CNV beyond the WAGR locus, as evidenced in our study by the characterization of two large deletions of 11 and 14 Mb, respectively. Thus, a single tool combines the diagnostic capacity of various techniques (karyotyping, MLPA, FISH, commercial CMA and exon-arrays).

Our array-CGH also allowed a better definition of rearrangements and breakpoints on the 11p13 region that the standard MLPA analysis, as demonstrated in the four positive controls. Interestingly, all positive samples carried bigger deletions than previously observed by MLPA, thus leading to a more accurate diagnosis of these patients. Therefore, our strategy represents a more sensitive and alternative tool for genetic diagnosis of aniridia, WAGR and associated syndromes. Some of the extended deleted regions found by our array-CGH were not identified by a previous MLPA analysis due to the lack of specific probes in the design, as is the case of alternative exons for *PAX6*, such as exon 5a. Similarly, microdeletions affecting downstream regions of *PAX6* also could escape to MLPA detection, as observed in two patients of our series. This MLPA failure was likely due to a poor resolution level of the oldest versions of the MLPA, which did not contain any specific probe for these regions. However, even the most updated MLPA design includes only two probes for the *ELP4* gene and does not cover most of the 16 identified enhancers of *PAX6* [36]. Besides, we identified two MLPA false negative cases in patients carrying deletions that should have been detected by this technique.

Some of the chromosomal aberrations here identified are likely to be microscopically visible and detectable by karyotyping and FISH analysis. However, our study shows that CGH technology has some advantages over these conventional techniques for the study of anidiria, giving a more precise mapping of breakpoints and being less time-consuming. Both advantages are important to obtain an accurate molecular diagnostic of patients, mainly of newborns and children with isolated aniridia, in order to determine the risk of developing systemic anomalies (such as Wilms tumor, development delay or obesity). However, karyotype and FISH cannot be totally replaced by array-CGH. Similarly to other dosage sensitive technologies, microarrays cannot detect balanced translocations or any other chromosomal rearrangement not involving loss or gain of genetic material. This was the case of a sporadic aniridia patient carrying an apparently balanced translocation tr(6,11), for which we could not identify any microarrangements at the chromosomal breakpoints by using our high-resolution custom CGH array.

Using WAGR-array was especially relevant for a correct clinical and molecular diagnosis of a child with aniridia and developmental delay, for which a WAGR-associated deletion had been falsely discarded by MLPA analysis. Instead, array-CGH analysis identifed a 11Mb microdeletion, leading to the diagnostic of a WAGRO syndrome before clear features of this syndrome had developed. This fact might prevent obesity in this child and improve the management of this patient. Besides, it is crucial to know not only the accurate extent of deletions and the implication of additional genes but also the precise genomic position of breakpoints for WAGR syndrome and other related-aniridia syndromes. In WAGR associated-deletions, a relationship between the deletion size and the risk of Wilms tumor has been described [37]. Furthermore, microdeletions with breakpoints located within the downstream region of *WT1* could cause renal pathology in adulthood [38]. Thus, array-CGH allows for the identification of putative contiguous gene syndromes and leads to an accurate diagnosis and an individualized follow up of patients with aniridia. Altogether, we consider that array-CGH is useful as a diagnostic tool and that its use should be encouraged.

Patients with ocular malformations are increasingly being analyzed by using CMAs, allowing to detect pathogenic CNVs involving a previously ocular-related gene in 8% of patients [21]. Although WAGR-array did not show chromosomal alterations on our cohort of patients with other ocular malformations, interestingly it allowed us to discard the risk of developing *WAGR*-associated anomalies, such as Wilms tumor, which is important in the management of syndromic and newborn patients. Similarly, WAGR-array was unable to identify *PAX6* defects in nine of our uncharacterized subjects with aniridia. These findings also suggest that there may be involved novel mutational mechanisms still unknown and/or undiscovered *loci* responsible for aniridia. In this sense, mutations and CNVs in *FOXC1* and *PITX2*, both genes responsible of Axenfeld-Rieger syndrome, could perhaps explain some rare cases of aniridia and aniridia-like phenotypes [39–42]. Thus, it is possible that the inclusion of additional *loci* in custom gene panels for array-CGH or next-generation sequencing could effectively identify novel mutations in these uncharacterized patients.

In conclusion, this work represents the largest series of patients with aniridia and related diseases analyzed by a targeted high-resolution oligonucleotide array. Our results emphasize the benefits of using a customized array-CGH (*versus* commercial CMA versions and MLPA) for improving diagnosis of aniridia and related syndromes, allowing not only for a rapid, reliable and powerful identification of copy number abnormalities at 11p13, but also for a more precise delimiting of boundaries. A correct detection of these genetic defects has important consequences for prognostic, management, follow-up and genetic counseling of patients and families with aniridia, particularly for syndromic patients and sporadic cases.
